# Octanoate Alleviates Dietary Soybean Oil-Induced Intestinal Physical Barrier Damage, Oxidative Stress, Inflammatory Response and Microbial Dysbiosis in Large Yellow Croaker (*Larimichthys Crocea*)

**DOI:** 10.3389/fimmu.2022.892901

**Published:** 2022-06-29

**Authors:** Zhou Zhang, Yuhang Tang, Wei Fang, Kun Cui, Dan Xu, Guobin Liu, Shuyan Chi, Beiping Tan, Kangsen Mai, Qinghui Ai

**Affiliations:** ^1^Key Laboratory of Aquaculture Nutrition and Feed (Ministry of Agriculture and Rural Affairs) and Key Laboratory of Mariculture (Ministry of Education), Ocean University of China, Qingdao, China; ^2^Laboratory of Aquatic Nutrition and Feed, College of Fisheries, Guangdong Ocean University, Zhanjiang, China; ^3^Laboratory for Marine Fisheries Science and Food Production Processes, Qingdao National Laboratory for Marine Science and Technology, Qingdao, China

**Keywords:** octanoate, intestinal health, dietary soybean oil, oxidative stress, inflammatory response, intestinal microbiota, acetic acid

## Abstract

Octanoate is a type of classical medium-chain fatty acids, which is widely used to treat neurological and metabolic syndrome. However, the specific role of octanoate in repairing intestinal health impairment is currently unknown. Therefore, we investigated whether dietary octanoate repaired the intestinal damage induced by surplus soybean oil in *Larimichthys crocea*. In this study, dietary octanoate alleviated abnormal morphology of the intestine and enhanced expression of ZO-1 and ZO-2 to improve intestinal physical barrier. Further, dietary octanoate increased antioxidant enzymic activities and decreased the level of ROS to alleviate the intestinal oxidative stress. Dietary octanoate also attenuated the expression of proinflammatory cytokines and the polarity of macrophage to reduce the intestinal inflammatory response. Moreover, the result of intestinal microbial 16S rRNA sequence showed that dietary octanoate repaired the intestinal mucosal microbial dysbiosis, and increased the relative abundance of *Lactobacillus*. Dietary octanoate supplementation also increased the level of acetic acid in intestinal content and serum through increasing the abundance of acetate-producing strains. Overall, in *Larimichthys crocea*, dietary octanoate might alleviated oxidative stress, inflammatory response and microbial dysbiosis to repair the intestinal damage induced by surplus soybean oil. This work provides vital insights into the underlying mechanisms and treatment strategies for intestinal damage in vertebrates.

## Highlight:

• Octanoate alleviated the soybean oil-induced intestinal physical barrier damage.
• Octanoate suppressed the oxidative stress and inflammatory response in the intestine.
• Octanoate repaired the intestinal microbial dysbiosis and increased the abundance of probiotic.
• Octanoate increased the level of acetic acid in intestinal content to benefit the intestinal health.

## Introduction

Dietary lipid is primarily emulsified, hydrolyzed, and ingested in the intestine (1). However, unhealthy dietary lipid patterns, such as surplus soybean oil (SO) are harmful, and will induce intestinal damage (2, 3). Severe intestinal damage even can induce and/or aggravate inflammatory bowel disease (IBD) and other metabolic syndromes (4). Though several studies on lipid-induced intestinal damage are available (1, 5), safer and more efficient nutritional therapy for repairing dietary lipid-induced intestinal injury remains an urgent requirement.

A number of functional lipids that improve intestinal damage have been identified to date, including short-chain fatty acids (SCFAs), medium-chain fatty acids (MCFAs), and n-3 polyunsaturated fatty acids (PUFAs) (1). Octanoate (C8:0) is a type of classical MCFA, which has special characteristics different from long-chain fatty acids (6). Firstly, octanoate could freely enter cells and be rapidly oxidated by mitochondria in a carnitine-independent manner. On account of its metabolic peculiarity, the ketogenic diet which is rich in octanoate, is employed worldwide to treat neurological and metabolic disorder (7). Secondly, octanoate could reduce the oxidative stress and inflammatory response. In zebrafish, octanoate ameliorated rotenone-induced oxidative stress and inflammatory response in the gut-brain axis (8). In mice, octanoate also inactivated Toll-like receptors (TLRs) to reduce the inflammatory response (9). Thirdly, octanoate could pass through the bacterial cytoderm freely as an anionic surfactant, destroy bacterial DNA duplication and inhibit lipase synthesis. Thus, octanoate will lead to microbial death of several pathobionts, such as *Cronobacter* (10), *Escherichia coli*, and *Staphylococcus aureus* (11). In mice, octanoate has further been shown to modulate the gut microbiota (12). Numerous studies also highlighted the dysbiosis of intestinal microbiota promoted the process of intestinal oxidative stress and inflammatory response (13, 14).

The previous study indicated that dietary octanoate could treat neurological and metabolic syndrome (15). However, to our knowledge, no reports have focused on the specific role of octanoate in repairing dietary lipid-induced intestinal damage to date. Hence, we investigated whether dietary octanoate repaired the intestinal damage induced by surplus SO. Fish represent a suitable model for investigating dietary lipid-induced intestinal damage because of their evolutionarily conserved nutrient-sensing systems (16). Earlier research by our group also demonstrated that dietary SO induced intestinal inflammation in large yellow croaker (*Larimichthys crocea*) (17). In this study, the potential benefits of octanoate in alleviating intestinal physical barrier damage, oxidative stress, inflammatory response, and microbial dysbiosis were evaluated. Our results may be extrapolated to develop efficient nutritional therapeutic strategies for the prevention and treatment of vertebrate intestinal disorder.

## Materials and Methods

### Diets and Animals

Six groups of diets containing equal amounts of protein (45.01%) and lipid (12.55%) were formulated for large yellow croakers (18). The negative control diet was formulated with 7% fish oil as the only lipid source (FO group). The positive control diet (SO group) and treatment diets were formulated with 7% soybean oil as the only lipid source. Based on the positive control diet, graded levels of 0.7 g/kg, 2.1 g/kg, 6.3 g/kg, and 18.9 g/kg sodium octanoate (purity: ≥ 99%, Sigma, USA) were added at the expense of microcrystalline cellulose to formulate other treatment diets **(** **Supplementary Table 1****)**. The Gas chromatograph-Mass spectrometer (GC/MS) was used to analyze the fatty acid profiles and corresponding levels of octanoate in six diets **(** **Supplementary Table 2****)**.

The experimental protocol was similar to those previously adopted in our laboratory (**Supplementary Figure 1**), and the feeding experiment was conducted at FuFa Aquatic Products Co., Ltd. (Ningde, China) (19). In brief, large yellow croakers were obtained from FuFa Aquatic Products Co., Ltd. (Ningde, China), and both sexes were used in the study. Prior to the start of the experiment, large yellow croakers were reared in floating sea cages (3 m × 3 m × 3 m) for 2 weeks to acclimate to the experimental diets and environment. At the start of the experiment, the fish were fasted for 24 h and weighed after being anesthetized with MS-222 (1:10000, Sigma, USA). Fish of similar sizes (13.00 ± 0.10 g) were randomly distributed into 18 sea cages (1 m × 1 m × 1.5 m). Each cage was stocked with 60 fish, and each diet was randomly assigned to triplicate cages. Fish were hand-fed to apparent satiation twice (05:00 and 17:00) daily for 10 weeks. During the experimental period, the temperature ranged from 19.5 to 25.5°C, the salinity from 25 to 28‰ and dissolved oxygen content was approximately 7 mg/L.

### Sampling and Dissection

At the end of the feeding experiment, MS-222 was prepared to anesthetize large yellow croakers which had been starved for 24 h. Firstly, blood was obtained from fish and clotted at 4°C for 4 h, the blood was centrifuged at 2, 500×g (10 min) to collect serum after that. Secondly, ten fish from each cage were dissected to obtain intestinal tissue and content. The intestinal content was directly stored in 1.5 mL Eppendorf tubes, and the intestinal tissue were stored in 1.5 mL Eppendorf tube after being washed by phosphate-buffered saline (PBS) (Biological Industries, Israel). Thirdly, to create a sterile environment, the surface of three fish per cage was sterilized with 75% alcohol. Then, the operation of sampling intestinal content and tract was conducted under the alcohol burner. The sample of intestinal content and tract was obtained and placed into 2 mL sterile cryopreservation tubes for flora analysis. Fourthly, the intestine of six fish was randomly collected from each cage, washed with PBS, preserved in 4% paraformaldehyde for 24 hours, and transferred to 75% alcohol for hematoxylin and eosin (H&E) staining. Lastly, these samples of serum, intestine, intestinal content, and tract were frozen in liquid nitrogen, then stored at −80°C for subsequent analysis. Meanwhile, the sample for H&E staining was stored at room temperature.

### Intestinal Histology Analysis

Intestinal morphology was determined based on the method described by the published paper (20). Briefly, fixed intestinal tissue was embedded in paraffin before staining. Then, paraffin sections were washed and stained with hematoxylin (Sigma, USA) for 10 min leading to nuclear staining. Paraffin sections were decolorized with HCl-alcohol for 12 s and incubated with eosin (Sigma, USA) for 3 min to stain the cytoplasm. Intestinal morphology was detected in the section under a microscope (ECLIPSE 80i, Nikon, Japan).

### RNA Extraction, cDNA Synthesis, and Real-Time Quantitative PCR (RT-qPCR)

The procedure of RNA and cDNA generating followed the previous study (19). Trizol reagent (Vazyme Biotech Co., Ltd, China) was added into the powdery intestinal tissue to extract the total RNA following the manufacturer’s protocol. The denaturing agarose gel (1.2%) and a NanoDrop spectrophotometer (Thermo Fisher Scientific, USA) were applied respectively to assess the quality and concentration of total RNA. Primers were designed **(** **Supplementary Table 3****)** based on the nucleotide sequences or referred in our previous study. The threshold of primers amplification efficiencies ranged between 0.95 and 1.05. β-actin was used as a reference gene in this study. The amplification was performed in a total volume of 20 μL containing 4 μL of cDNA, 0.5 μL of each primer, 10 μL of ChamQ Universal SYBR qPCR Master Mix (Vazyme Biotech Co., Ltd, China), and 5 μL of RNase-free water. PCR cycling conditions were 10 s at 95°C, 10 s at 58°C and 20 s at 72°C for 39 cycles. Melting curve analysis was carried to confirm that a single PCR product was present. RT-qPCR was carried out on a CFX96 Real-Time PCR Detection System (BIO-RAD, USA), and the relative gene expression levels were calculated with the 2^–ΔΔCT^ method (21).

### Enzymatic Activity Assays

PBS was precooled to serve as the medium to homogenize the intestine. Then the supernatant was extracted after centrifuging the homogenate at 2, 500×g for 15 min. According to the method described by Yin et al. (20), brush border membranes (BBM) were purified from the homogenate of the intestine.

The method of measuring the activities of superoxide dismutase (SOD), peroxidase (POD), catalase (CAT), total antioxidant capacity (T-AOC), trypsin, lipase (LPS), α-amylase (α-AMS), alkaline phosphatase (AKP), acid phosphatase (ACP), and the levels of endothelin-1 (ET-1), D-lactic acid (D-Lac), reduced glutathione (GSH), malondialdehyde (MDA), complement 3 (C3) was referred to the instruction of commercial kit (Nanjing Jiancheng Bioengineering Institute, China). Besides, the kit for measuring ET-1, D-Lac, and C3, was commercial ELISA kit. The activity of leucine-aminopeptidase (LAP) was determined according to the description of Liu et al. (22). In brief, leucine-p-nitroanilide (Sigma, USA) servicing as substrate was incubated with 100 μL supernatant fraction (37°C) and phosphate buffer. The absorbance of samples was continuously monitored in Multiskan Spectrum (Thermo, USA) at 405 nm for 15 min.

### Western Blot Analysis

The western blotting procedure followed the previous study (23). Intestinal tissue was lysed by RIPA reagent (Solarbio, China) with protease inhibitors and phosphatase inhibitors and centrifuged at 12, 000×g for 10 min to obtain supernatant. Protein concentration was determined by a BCA Protein Assay Kit (Beyotime Institute of Technology, China). An equal amount of 20 μg protein samples was separated by 10% sodium dodecyl sulphate polyacrylamide gel electrophoresis (SDS-PAGE) and transferred to activated 0.45 μm polyvinylidene difluoride (PVDF) membranes (Pall Corporation, USA). The proteins on PVDF membranes were blocked with 5% nonfat dry milk in tris-buffered saline with Tween™ (TBST) at room temperature for 2 h. Then the PVDF membranes were incubated with primary antibodies overnight at 4°C. Subsequently, membranes were incubated with secondary antibodies for 2 h after membranes were washed with TBST. At last, immune complexes were visualized using Beyo ECL Plus Kit (Beyotime Institute of Technology, China).

Anti-glyceraldehyde 3-phosphate dehydrogenase (GAPDH, 1:5000 dilution; Golden Bridge Biotechnology, China) was used as the reference. Anti-cluster of differentiation 68 (CD68, 1:2000 dilution), anti-cluster of differentiation 209 (CD209, 1:1000 dilution), anti-zonula occludens-1 (ZO-1, 1:1000 dilution);, and anti-zonula occludens-2 (ZO-2, 1:500 dilution) were obtained from Abcam (England). Anti-macrophage mannose receptor 1(MRC1, 1:200 dilution) was obtained from Sangon (China).

### Fatty Acids Analysis

The method of fatty acid profiles analysis was based on the procedure described by Li et al. (19) with some modifications. Solid samples (intestinal tissue and experimental diets) were freeze-dried, then approximately 100 mg were collected and placed in 3 mL KOH–ethanol (1 N) (Shanghai yuanye Bio-Technology Co., Ltd, China) to saponify total lipids. Fatty acids were esterified after acid-catalyzed methylation with 3 mL methanolic hydrogen chloride (2 N) (Shanghai yuanye Bio-Technology Co., Ltd, China). Fatty acid methyl esters (FAME) were extracted and purified by chromatographic grade n-hexane (Shanghai yuanye Bio-Technology Co., Ltd, China), and quantified by a Gas chromatograph-Mass spectrometer (GC/MS) (Agilent Technologies Inc., Santa Clara, California, USA).

In the measurement of acetic acid, the preparation of FAME was similar to the above, except that, 20 μL serum or 200 μL intestinal content was saponified and methylated directly without freeze-drying. GC/MS was also used to quantify the FAME of acetic acid.

### Culture and Treatment of Intestinal Cells

The method of obtaining intestinal cells was similar to Fang et al. (24). Briefly, 15% fetal bovine serum (FBS) (Biological Industries, Israel) was added into Dulbecco’s modified Eagle’s medium (DMEM)/F12 (Biological Industries, Israel) to culture intestinal cells isolated from healthy large yellow croakers. Cells were cultivated in 27°C and 5% CO_2_ atmosphere. Intestinal cells were seeded in six-well plates, and after 48 h of culture, intestinal cells were serum-starved with FBS-free DMEM/F12 for 2 h. When intestinal cells completed hungry, different concentrations of docosahexaenoic acid (DHA) (Sigma, USA), linoleic acid (LA) (Sigma, USA), and octanoate (Sigma, USA) were supplied in DMEM/F12 individually or together to culture intestinal cells to simulate the situation *in vivo*. Then, cells were harvested for further analysis.

### Determination of ROS Generation

The change of intracellular reactive oxygen species (ROS) was determined according to the accompanying instructions of the reactive oxygen species detection kit (Beyotime Institute of Technology, China) and Fang et al. (25). In brief, cells were cultured in 96-well plates with DMEM/F12, stimulated with fatty acids for 12 h, then loaded with cell-permeable 2’, 7’ dichlorofluorescein diacetate (DCFH-DA). DCFH-DA was prepared into 10 μmol/L by serum-free DMEM/F12 medium. The original medium was discarded, and cells were washed with PBS and added with the prepared DCFH-DA. After incubation at 27°C for 20 min, cells were washed three times with DMEM/F12 medium to remove the remaining DCFH-DA. Multiskan Spectrum (Thermo, USA) was used to detect the fluorescence intensity at an excitation wavelength of 488 nm and an emission wavelength of 535 nm.

### Intestinal Microbiota Sequencing and Analysis

The protocol of intestinal microbiota sequencing and analysis was similar to our previous study (20). Total genomic DNA of intestinal content flora and mucosal microbiota in each group was extracted (26) and the V3-V4 region of the bacterial 16S rRNA gene was amplified by PCR using the 515f/907r primer (Fwd5’-GTGCCAGCMGCCGCGGTAA-3’, Rev5’-CCGTCAATTCCTTTGAGTTT-3’) with the barcode. Subsequently, sequencing was performed on an Illumina MiSeq platform, provided by Beijing Novogene Genomics Technology Co. Ltd. (China). Complete data were submitted to the NCBI Sequence Read Archive (SRA) database under accession number PRJNA814750.

FLASH (V1.2.7, http://ccb.jhu.edu/software/FLASH/) was used to merge reads from the same original DNA (27). Uparse software (Uparse v7.0.1001, http://drive5.com/uparse/) was applied to cluster the unique sequences to acquire operational taxonomic units (OTUs) according to the similarity of sequence distance based up to 97% or greater (28). Subsequently, the representative OTUs were annotated through the RDP Classifier (Version 2.2, http://sourceforge.net/projects/rdp-classifier/ and GreenGene database (http://greengenes.lbl.gov/cgi-bin/nph-index.cgi (29). QIIME (Quantitative Insights Into Microbial Ecology) V1.7.0 software package (http://qiime.org/index.html and the UPARSE (http://drive5.com/uparse/) pipeline were adopted to analyze the alpha and beta diversity. Principal coordinates analysis (PCoA) was conducted with Mothur and R software packages (http://www.R-project.org). Linear discriminant analysis (LDA) effect size (LefSe) analysis was used to identify the different abundant taxa among groups (30).

### Calculations and Statistical Analysis

The perimeter ratio (PR) = the total length of intestinal epithelium/the perimeter of the intestine.

The total length of intestinal epithelium and the perimeter of the intestine were measured by the Image J software (USA). The villus height (VH) and muscular thickness (MT) were measured by the Image-Pro Plus version 6.0.0.260 (USA).

All statistics were evaluated by SPSS 19.0 (IBM, USA). All data were subjected to a one-way analysis of variance (ANOVA) and followed by Tukey’s multiple range test or independent t-tests. Bars bearing the different letter were significantly different among treatments (*P < 0.05), and results were presented as means ± S.E.M. (standard error of the mean).

## Results

### Octanoate Supplementation Alleviated Dietary SO-Induced Intestinal Physical Barrier Damage and Digestive Capacity Decrease

**Figure 1:**
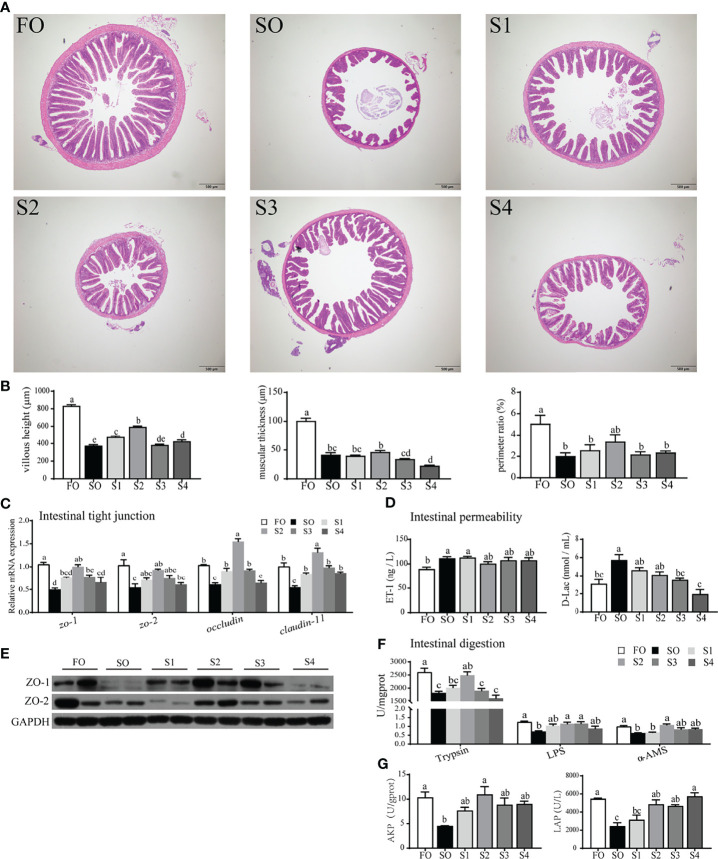
Octanoate supplementation alleviated dietary SO-induced intestinal physical barrier damage and digestive capacity decrease. Histopathological changes in the intestine tissue from the experimental groups were observed by H&E staining **(A)** and assessed by indices of villus height (VH), muscular thickness (MT), and perimeter ratio (PR) **(B)**. The relative mRNA expression of tight junction protein (*zo-1*, *zo-2*, *occludin*, and *claudin-11*) in the intestinal tissue were measured **(C)**. The protein expression of tight junction protein (ZO-1 and ZO-2) in the intestinal tissue were measured (n = 2) **(E)**. The level of ET-1 and D-Lac in the serum were measured **(D)**. The enzymic activities of Trypsin, LPS, and α-AMS in the intestinal tissue **(F)**, and LAP, AKP in intestinal brush margin were measured **(G)**. Data were presented as means ± S.E.M. Means in each bar sharing the same superscript letter or absence of superscripts were not significantly different determined by Tukey’s test (*P* ≥ 0.05). S.E.M.: standard error of means (n = 3).

The intestinal physical barrier serves as ‘mechanical defense system’ protecting the intestine. In this study, the intestinal physical barrier was disrupted by surplus SO, while dietary octanoate supplementation alleviated the adverse effect (**Figures 1A–E)**. Specifically, compared to the SO group, dietary octanoate increased the VH, MT, and PR of intestinal tissue, and only VH showed a significant increase in group S2 (*P* < 0.05) (**Figures 1A, B**). A number of indices of intestinal tight junction (TJ) proteins and permeability were further tested to assess the integrity of the intestinal physical barrier. The result suggested that dietary octanoate supplementation significantly increased the mRNA expression of *zo-1*, *zo-2*, *occludin*, and *claudin-11* in group S2 relative to group SO (*P* < 0.05) (**Figure 1C**). Consistent with mRNA levels, relative protein levels of ZO-1 and ZO-2 were both enhanced in group S2 **(** **Figure 1E**). Notably, the D-Lac level in groups S3 and S4 showed a marked decline compared to the group SO (*P* < 0.05), while the ET-1 level in treatment groups showed no significant change compared to the group SO (*P* > 0.05). (**Figure 1D**).

Excess dietary SO had an inhibitory effect on intestinal digestion (**Figures 1F, G**). Presently, compared with SO group, octanoate supplementation conspicuously boosted digestive enzymic activities in group S2 (*P* < 0.05), including trypsin, LPS, and α-AMS (**Figure 1F**). Activities of LAP and AKP in intestinal BBM were also considerably enhanced in the group S2 compared to the SO group (*P* < 0.05) (**Figure 1G**). Above all, these data indicated that, dietary octanoate clearly improved the intestinal physical barrier and digestion which were damaged by SO.

### Octanoate Supplementation Reduced Dietary SO-Induced Intestinal Oxidative Stress and Inflammatory Response

**Figure 2:**
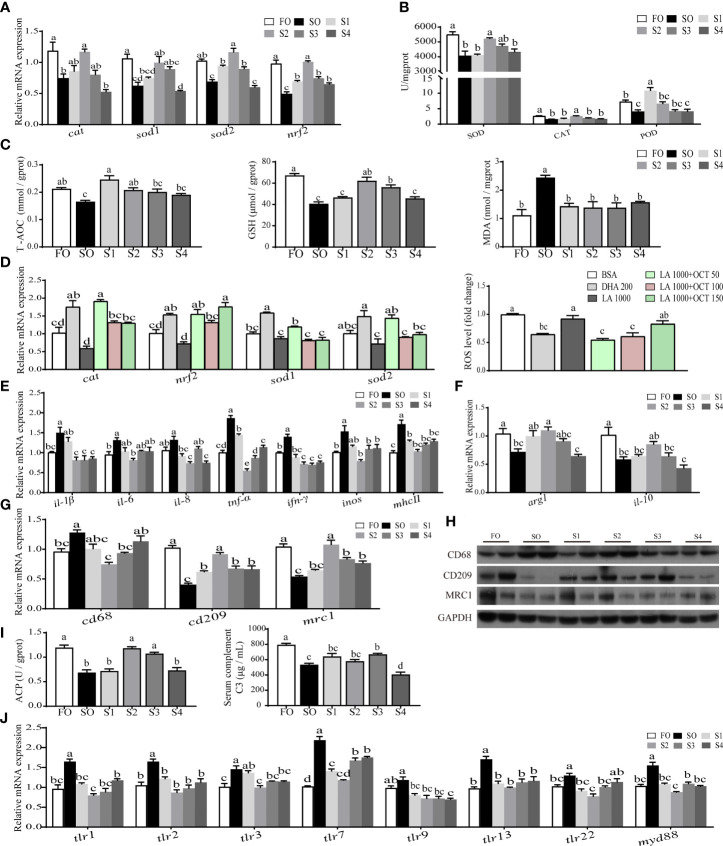
Octanoate supplementation reduced dietary SO-induced intestinal oxidative stress and inflammatory response. The relative mRNA level of key antioxidation-related genes (*cat*, *nrf2*, *sod1*, and *sod2*) in the intestinal tissue were measured **(A)**. The activities of SOD, CAT, and POD in the intestinal tissue were measured **(B)**. The level of T-AOC, MDA, and GSH in the intestinal tissue were tested **(C)**. The relative mRNA expression of key antioxidation-related genes (*cat*, *nrf2*, *sod1*, and *sod2*) and the fold change of ROS in treated intestinal cells were measured **(D)**. The relative mRNA expression of proinflammatory cytokines (*il-1β*, *il-6*, *il-8*, *tnf-α*, *ifn-γ*, *inos*, and *mhcⅡ*) **(E)** and anti-inflammatory cytokines (*arg1* and *il-10*) **(F)** in the intestinal tissue were measured. The relative mRNA **(G)** and protein (n = 2) **(H)** level of CD68, CD209, and MRC1 in the intestinal tissue were measured. The activity of ACP in the intestinal tissue and the level of serum C3 were measured **(I)**. The relative mRNA expression of *tlr1*, *tlr2*, *tlr3*, *tlr7*, *tlr9*, *tlr13*, *tlr22*, and *myd8*8 in the intestinal tissue were measured **(J)**. Data were presented as means ± S.E.M. Means in each bar sharing the same superscript letter or absence of superscripts were not significantly different determined by Tukey’s test (*P* ≥ 0.05). S.E.M.: standard error of means (n = 3).

To further describe the intestinal environment completely, relative indices of oxidative stress (**Figures 2A–D**) and inflammatory response (**Figures 2E–J**) were measured respectively.

On the one hand, the result indicated that the intestinal oxidative stress induced by SO was reduced by dietary octanoate supplementation. Specifically, the mRNA expression of *cat*, *sod1*, *sod2*, and *nrf2* were markedly increased in group S2 relative to group SO (*P* < 0.05) (**Figure 2A**). Compared to the SO group, dietary octanoate markedly enhanced antioxidant enzymatic activities in S2 group (*P* < 0.05) (**Figure 2B**), including SOD, CAT, and POD. Meanwhile, in S2 group, non-enzymatic antioxidant indices (including the level of T-AOC and GSH) were also improved, and the MDA level significantly lessened (*P* < 0.05) (**Figure 2C**). With the support of the data of fatty acid profiles in intestinal tissue (**Supplementary Table 4**) and pre-experiment in intestinal cells (**Supplementary Figures 2A, B**), *in vitro*, we decided to use 1000 μM LA incubating cells contrast to 200 μM DHA incubating cells to simulate intestinal cells *in vivo*. We found that 50 μM octanoate markedly diminished the 1000 μM LA-induced ROS production in intestinal cells (*P* < 0.05) (**Figure 2D**).

On the other hand, the result also showed that the intestinal inflammatory response induced by SO was alleviated by dietary octanoate supplementation. In this study, the mRNA expression of several proinflammatory cytokines (including *il-1β*, *il-6*, *il-8*, *tnf-α*, *ifn-γ*, *inos*, and *mhcⅡ*) prominently declined in the group S2 compared with SO group (*P* < 0.05) (**Figure 2E**). Conversely, dietary octanoate facilitated the expression of anti-inflammation cytokines (*arg1* and *il-10*) in the group S2 (**Figure 2F**). CD68, CD209, and MRC1 were known biomarkers of macrophage polarization. Compared to the SO group, dietary octanoate decreased the mRNA and protein expression of CD68. Moreover, dietary octanoate also increased the mRNA and protein expression of CD209 and MRC 1 (**Figures 2G, H**). Intestinal ACP activity and serum C3 level had a marked increase in groups S3 compared to the group SO (*P* < 0.05) (**Figure 2I**). We previously verified that dietary SO induced inflammation through TLRs activation (31). In this study, relative to the group SO, octanoate supplementation lowered the relative mRNA expression of TLRs-related genes (*tlr1*, *tlr2*, *tlr3*, *tlr7*, *tlr9*, *tlr13*, *tlr22*, and *myd8*8) in group S2 (**Figure 2J**). These collective results suggested that dietary octanoate reduced intestinal oxidative stress and inflammatory response induced by excess SO.

### Octanoate Supplementation Modulated Intestinal Mucosal Microbiota

Intestinal mucosal microbiota represents the body’s first line of defense against pathogenic microorganisms. 16S ribosomal RNA sequencing of intestinal mucosal microbiota was conducted to evaluate the regulatory effects of octanoate in the intestinal mucosal microbiota. In this study, 1, 575, 056 high-quality sequences were obtained from 24 mucosal samples with 65, 019.5 ± 4938.5 sequences per sample. After being assembled, quality screened, and trimmed, the study resulted in the identification of 1, 216 OTUs with 97% sequence similarity level among samples (data not shown). For all samples, both the rarefaction curve and species accumulation boxplot, tended to approach the saturation plateau (**Supplementary Figures 2C, D**), indicative of the completeness and depth of sequencing efforts.

**Figure 3:**
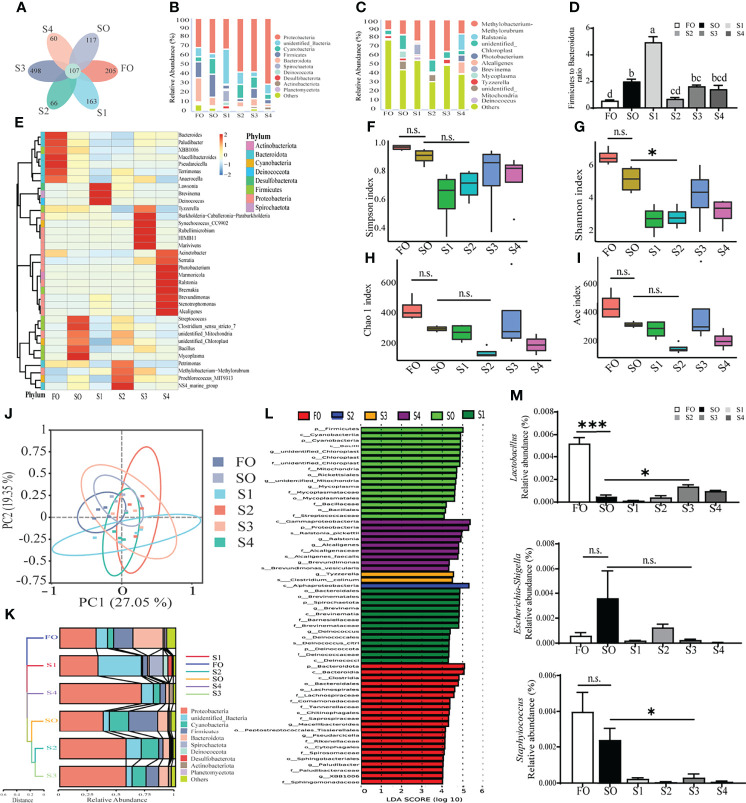
Octanoate supplementation modulated intestinal mucosal microbiota. The profile of gut mucosal microbiota (n = 4). Venn diagram **(A)**, the histogram of relative abundance of phylum **(B)** and genus **(C)** (Only top 10 most relative abundant bacterial phyla and genera were shown. Other phyla and genera were all assigned as ‘Others’.), heatmap comparison and hierarchical clustering dendrogram based on the relative abundance of dominant bacterial genera at the phylum and genus levels **(E)** and the ratio of Firmicute to Bacteroidetes (n = 3) **(D)** were calculated to portray the fundamental structure of mucosal microbiota. Alpha diversity indices included the Simpson index **(F)**, the Shannon index **(G)**, the Chao 1 index **(H),** and the abundance-based coverage estimator (Ace) index **(I)** were calculated to compare the community diversity and richness. The beta diversity analyses were performed on the weighted Unifrac distance matrix to analyze the extent of similarities in microbial communities, including the principal coordinates analysis (PCoA) **(J)** and UPGMA clustering **(K)**. Linear discriminant analysis Effect Size (LEfSe) analysis **(L)** was conducted to find out the biomarker with noteworthy differences between groups. The relative abundance of *Lactobacillus*, *Escherichia-Shigella*, and *Staphylococcus* settling on the mucosa were calculated (n = 3) **(M)**. Results of alpha diversity indices and the relative abundance of *Lactobacillus*, *Escherichia-Shigella*, and *Staphylococcus* were analyzed using independent t-test (n.s., No significant; *P* ≥ 0.05, *0.01 *≤ P* < 0.05, ***P < 0.001).

A Venn diagram showed that 107 OTUs were shared by all groups. The number of unique OTUs in FO, SO, S1, S2, S3, and S4 groups were 205, 117, 163, 66, 498, and 60, respectively (**Figure 3A**). A histogram depicting relative abundance of phylum and genus reveals that Proteobacteria, unidentified Bacteria, and Cyanobacteria were detected as predominant bacterial phyla in the mucosal microbiota among all groups at the phylum level (**Figure 3B**). *Methylobacterium-Methylorubrum*, *Ralstonia*, and unidentified *Chloroplast* were detected as the richest bacterial genera in the mucosal microbiota among all groups at the genus level (**Figure 3C**). Further analysis of the mucosal microbiota with heatmap comparison and hierarchical clustering dendrogram demonstrated that octanoate induced alterations in the dominant bacterial composition (**Figure 3E**). Though the Firmicute to Bacteroidetes ratio was conspicuously augmented in groups SO and S1 in contrast to the group FO, octanoate supplementation induced a considerable decrease in the S2 group compared to the group SO (*P* < 0.05) (**Figure 3D**).

Alpha diversity indices, including the Simpson index, the Shannon index, the Chao 1 index, and the abundance-based coverage estimator (Ace) index were calculated to compare the community diversity and richness. The result showed that all alpha diversity indices were decreased in group S2 relative to the group SO (**Figures 3F–I**). Moreover, beta diversity analysis was performed on the weighted Unifrac distance matrix to analyze the extent of similarities in microbial communities among groups. Principal coordinates analysis (PCoA) showed that the octanoate supplementation resulted in a more marked difference in the structure of intestinal mucosal microbiota between treatment groups and SO group, which explained 27.05% of the total variance observed in PC1 and 19.35% of the total variance observed in PC2 (**Figure 3J**). The hierarchical clustering tree illustrated mucosal microbiota structures from groups SO, S2, and S3 were similar and clustered within one lower branch, whereas those of groups FO, S1, and S4 were distinct and formed another branch (**Figure 3K**).

To identify the differentially abundant taxa among experimental samples, linear discriminant analysis effect size (LEfSe) analysis was conducted. The result revealed marked differences in taxonomic distribution of mucosal microbiota communities between control groups and treatment groups. Dietary SO induced a significant increase in the relative abundance of phylum Firmicutes, class Bacilli, order Mycoplasma, family Streptococcaceae, and genus *Cutibacterium*, while the octanoate promoted the relative abundance of class Alphaproteobacteria in the group S2, and the relative abundance of genus *Tyzzerella* and species *Clostridium colinum* were richened in the group S3 (**Figure 3L**).

Further in-depth investigation of the abundance of functional bacterial strains revealed significantly higher relative abundance of *Lactobacillus* in group S3 compared to the group SO (*P* < 0.05). Conversely, the relative abundance of *Escherichia-Shigella* and S*taphylococcus* were lower in group S3 than group SO (**Figure 3M**). Together, these findings strongly implied that adequate dietary octanoate supplementation modulated the overall structure of mucosal microbiota which was disturbed by SO, and facilitated the increased abundance of probiotics.

### Octanoate-Induced Changes in the Intestinal Microbiota had a Tight Correlation With Intestinal Repair

To investigate the correlation between the intestinal damage repair and the change of intestinal mucosal microbiota, Spearman’s correlation analysis was conducted.

**Figure 4:**
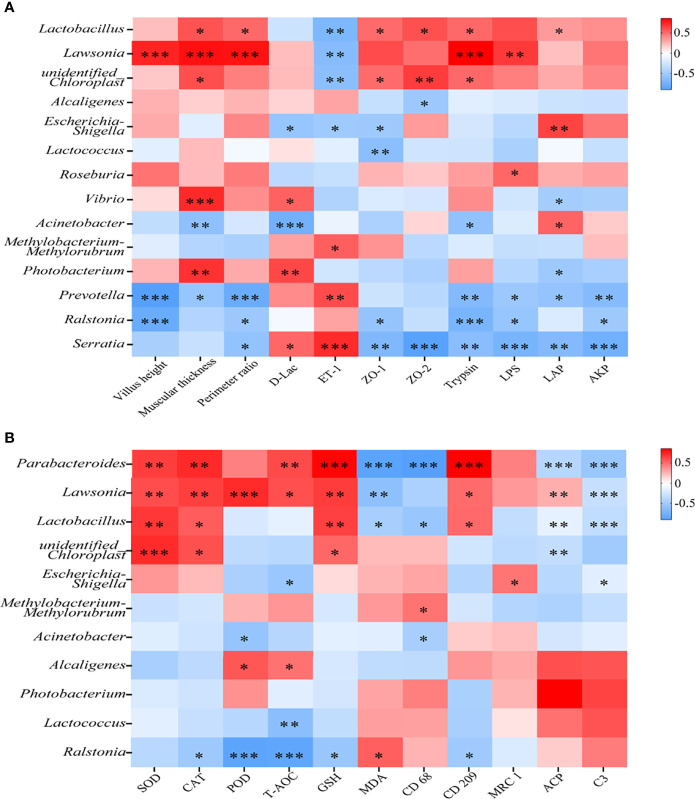
Octanoate-induced changes in the intestinal microbiota had a tight correlation with intestinal repair. The Spearman’s correlation coefficient between mucosal microbiota and intestinal physical barrier (intestinal VH, MT, PR, the level of D-Lac, ET-1, the relative protein level of ZO-1 and ZO-2), digestive capacity (Trypsin, LPS, α-AMS, LAP, and AKP) were calculated and the heatmap was exhibited **(A)**. The Spearman’s correlation coefficient between mucosal microbiota and intestinal antioxidative capacity (SOD, CAT, POD, and the content of T-AOC, GSH, MDA), and inflammatory response (the relative protein level of CD68, CD209, MRC1, intestinal ACP, and serum C3) were calculated and the heatmap was exhibited **(B)**, *0.01 ≤ *P* < 0.05, ** 0.001 ≤ *P* < 0.01, ***P < 0.001.

The correlation between physical barrier, digestion, and intestinal mucosal microbiota was presented in **Figure 4A**. Among the top 5 abundant genera, the change of *Alcaligenes* and *Methylobacterium-Methylorubrum* abundance was not remarkably correlated with physical barrier and digestion improvement, whereas the change of *Photobacterium*, *Ralstonia*, and unidentified *Chloroplast* abundance showed a significant correlation with these indices (*P* < 0.05). Heatmap analysis also showed significant positive association of *Lawsonia* and significant negative association of *Prevotella* with intestinal VH, MT, PR, trypsin, and LPS. Moreover, *Serratia* had a tight correlation with physical barrier and digestion.

The correlation between oxidative stress, inflammatory response, and intestinal mucosal microbiota was determined in **Figure 4B**. Almost all of the top 5 abundant genera showed weak correlations with indices of oxidative stress and inflammatory response. The change of *Lawsonia* and *Parabacteroides* abundance had a strongly negative correlation with oxidative stress and inflammatory response. Taken together, these results suggested that dietary octanoate might modulate the intestinal microbiota to promote the repair of intestinal damage.

### Octanoate Supplementation Up-Regulated Acetic Acid Production

**Figure 5:**
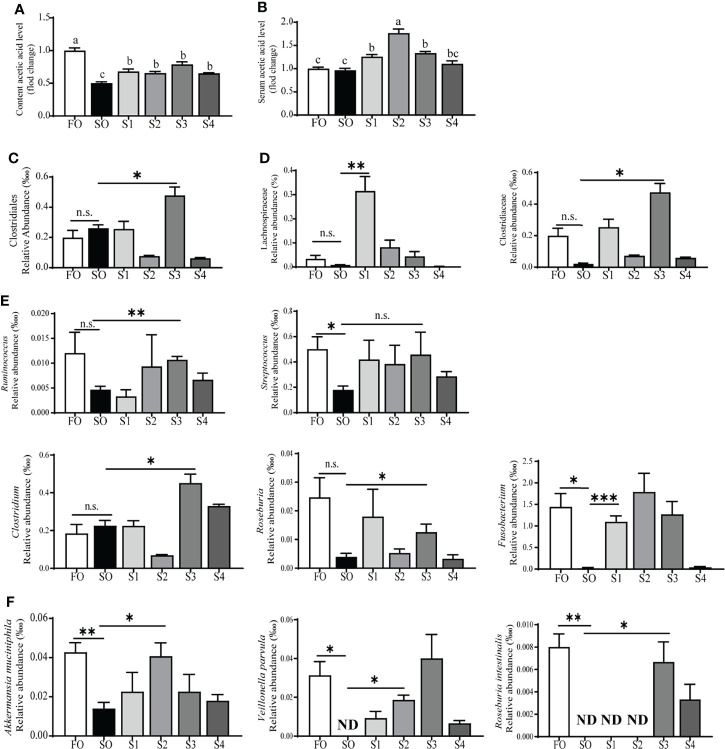
Octanoate supplementation up-regulated acetic acid production. The fold change of acetic acid level in intestinal content **(A)**, and serum **(B)**. The relative abundance of acetate-producing strains in content flora at the order **(C)**, family **(D)**, genus **(E)**, and species **(F)** level. ND, Not found in samples. Data of the fold change of acetic acid level were presented as means ± S.E.M. Means in each bar sharing the same superscript letter or absence of superscripts were not significantly different determined by Tukey’s test (*P* ≥ 0.05). S.E.M., standard error of means (n = 3). Data of the relative abundance of acetate-producing strains were analyzed using independent t-test (n.s., No significant; *P* ≥ 0.05, *0.01 *≤ P* < 0.05, ** 0.001 ≤ *P* < 0.01, ***P < 0.001) (n = 3).

According to the previous study (32), acetic acid is the most abundant SCFA in the intestinal content, and it was produced by the intestinal lumen content flora to benefit the intestine. Acetic acid fluctuation in the intestinal content and serum were detected by GC/MS. The result suggested that, compared to the group SO, supplementation with dietary octanoate significantly promoted acetic acid levels in both intestinal content and serum (*P* < 0.05) (**Figures 5A, B**). And the 16S ribosomal RNA sequencing of intestinal content flora was operated to explore the change of acetate-producing strains (**Supplementary Figures 2G–O**). In this study, both the rarefaction curve and species accumulation boxplot, tended to approach the saturation plateau (**Supplementary Figure 2E, F**), indicative of the completeness and depth of sequencing efforts.

Alpha diversity indices suggested that low-dosage octanoate diminished the community diversity while high doses reversed this effect (**Supplementary Figures 2J–M**). Beta diversity analyses were performed on the weighted Unifrac distance matrix. The result of PCoA showed that, octanoate intervention generated a more conspicuous difference in the structure of intestinal content flora between treatment groups and the SO group (**Supplementary Figure 2N**). The content flora structure of groups FO, S2, and S4 were similar and clustered within one lower branch, whereas that of group SO was distinct from them and formed another branch (**Supplementary Figure 2O**).

The result further indicated that the relative abundance of the bacteria producing acetic acid was increased (**Figures 5C–F**). Specifically, compared to the group SO, at the order level (**Figure 5C**), the relative abundance of Clostridiales in group S3 had a marked increase (*P* < 0.05); at the family level (**Figure 5D**), the relative abundance of Lachnospiraceae and Clostridiaceae showed a statistical augment (*P* < 0.05); at the genus level (**Figure 5E**), the relative abundance of *Ruminococcus*, *Clostridium*, and *Roseburia* in group S3 had a remarkable increase (*P* < 0.05), and the significant change of the relative abundance of *Fusobacterium* appeared in the group S1 (*P* < 0.05). *Streptococcus* showed a tendency to increase with dietary octanoate supplementation (*P* > 0.05). The supplementation of octanoate reverses the decrease of *Akkermansia muciniphila*, *Veillonella parvula*, and *Roseburia intestinalis* in groups S1, S2, and S3 compared to the group SO, respectively (**Figure 5F**) (*P* < 0.05). Overall, these findings indicated that dietary octanoate supplementation increased the relative abundance of acetate-producing strains and augmented the level of acetic acid to benefit intestinal health.

## Discussion

Previous studies have verified that unhealthy dietary lipid patterns can damage the intestinal health, such as increased intestinal permeability (33), lessened TJ protein level, excessive oxidative stress (34), and serious inflammatory response (35, 36). Octanoate has often been applied to remedy the neurological and metabolic syndrome, but the specific role of octanoate in the treatment of intestinal damage has not been established to date. Thus, in this study, the intestinal injury induced by SO was treated with dietary octanoate to investigate its specific role. Notably, compared to SO diet feeding, dietary octanoate supplementation ameliorated abnormal morphology of the intestine and enhanced protein expression of ZO-1 and ZO-2, in turn diminishing permeability. Dietary octanoate also enhanced intestinal digestion through increasing digestive enzyme activities. These results are in agreement with previous data obtained in piglet and mice (37, 38). Overall, the result clearly suggested that dietary octanoate effectively rescued the surplus SO-induced damage of intestinal physical barrier, increased the TJ protein level and improved the digestion.

Further, compared to SO diet feeding, dietary octanoate supplementation increased the intestinal antioxidative capacity and decreased ROS production, then alleviated the intestinal oxidative stress. These results are in agreement with previous data obtained in zebrafish (8) and rats (39). The study showed that octanoate could reduce real protonophoric uncoupling induced by long-chain fatty acids to lessen the production of ROS (6). On the other hand, dietary octanoate alleviated intestinal inflammatory response induced by SO through reducing the expression of proinflammatory genes and the polarity of macrophages. These results were consistent with data from previous studies on mice (40). The previous study indicated that activation of the anti-inflammatory state in macrophages could be attributed to octanoate-induced up-regulation of β-oxidation in macrophages (41). Moreover, the augmented ACP activity and C3 levels also corroborated the decrease in intestinal inflammatory response induced by dietary octanoate. Further, SO-induced mRNA expression of TLR-related genes was decreased with the supplementation of octanoate, supporting the theory that octanoate inactivated TLRs to reduce inflammation. This was similar to the result obtained with RAW246.7 cells (9). Based on the collective result, we conclude that dietary supplementation with octanoate could effectively reduce intestinal oxidative stress and inflammatory response induced by dietary surplus SO.

Accumulating evidence suggested that intestinal microbiota maintains intestinal homeostasis and host health, while microbial dysbiosis plays a role in pathogenesis of IBD (35, 42). In this study, dietary octanoate reduced the alpha diversity indices of intestinal mucosal microbiota. Though the lower alpha diversity indices were connected with the occurrence of IBD (12), the ratio of Firmicute to Bacteroidetes, another vital index of microbiota balance, was decreased (43). The structures of treatment groups microbiota were away from group FO on the weighted Unifrac distance matrix. We further investigated the correlation of specific genera and intestinal damage repairment by Spearman’s correlation coefficient. *Ralstonia*, isolated from cystic fibrosis patients (44), were negatively correlated with indices of antioxidant capacity (POD, T-AOC, and GSH), digestive enzymic activities (trypsin, LPS, and AKP), and anti-inflammatory capacity (IL-10 and CD209). The previous study proved *Alcaligenes* could serve as an immunologic adjuvant to stimulate dendritic cells in systemic vaccination (45), but little correlation with inflammatory response was found in this study. *Lawsonia* is a pathogen in swine (46), but some species may benefit fish because of the tight and positive correlation between *Lawsonia* and intestinal health in this study. Thus, the potential of *Lawsonia* as a fish probiotic should be further explored. In mice, the researchers also suggested that dietary octanoate benefited the intestinal health through modulating intestinal microbiota (38).

*Lactobacillus* was identified as probiotic, and *Escherichia-Shigella*, *Staphylococcus* species were identified as pathobionts. Octanoate promoted the abundance of *Lactobacillus* and reduced that of *Escherichia-Shigella* and *Staphylococcus*, similar to previous finding (15). We suggested that the octanoate-induced decrease in pathogenic bacteria was attributed to its ability on disrupting bacterial DNA replication and inhibiting lipase synthesis (15). The lower abundance of pathogenic bacteria might contribute to the increase of probiotics. Although high doses of octanoate might exert an excessive killing effect on bacteria, including both pathogenic bacteria and probiotics, octanoate-induced benefit for the intestinal microbiota was clearly evident. Overall, dietary octanoate repaired the intestinal mucosal microbial dysbiosis and there was a strong correlation between changes in intestinal mucosal microbiota and the repair of intestinal damage. Dietary octanoate might affect the intestinal health through modulating intestinal microbiota, but the underlying mechanisms need to be further explored in depth.

Intestinal microbiota not only resists invasion of antigens but also produces beneficial metabolites, such as acetic acids (13, 47, 48). The previous study showed that acetic acid alleviated high-carbohydrate induced intestinal inflammation by suppressing MAPK and NF-κB signaling pathways in Nile tilapia (49). The acetic acid level in the intestine and serum was increased upon dietary octanoate supplementation. The result was consistent with data from an earlier study on mice (12). The 16S ribosomal RNA sequencing of intestinal content flora showed that dietary octanoate augmented the relative abundance of acetate-producing strains (including *Ruminococcus*, *Clostridium*, *Roseburia*, and *Akkermansia muciniphila* et al.) (47). Notably, dietary octanoate enhanced the abundance of acetate-producing strains, subsequently promoting the acetic acid level to benefit the intestinal health.

## Conclusion

**Figure 6:**
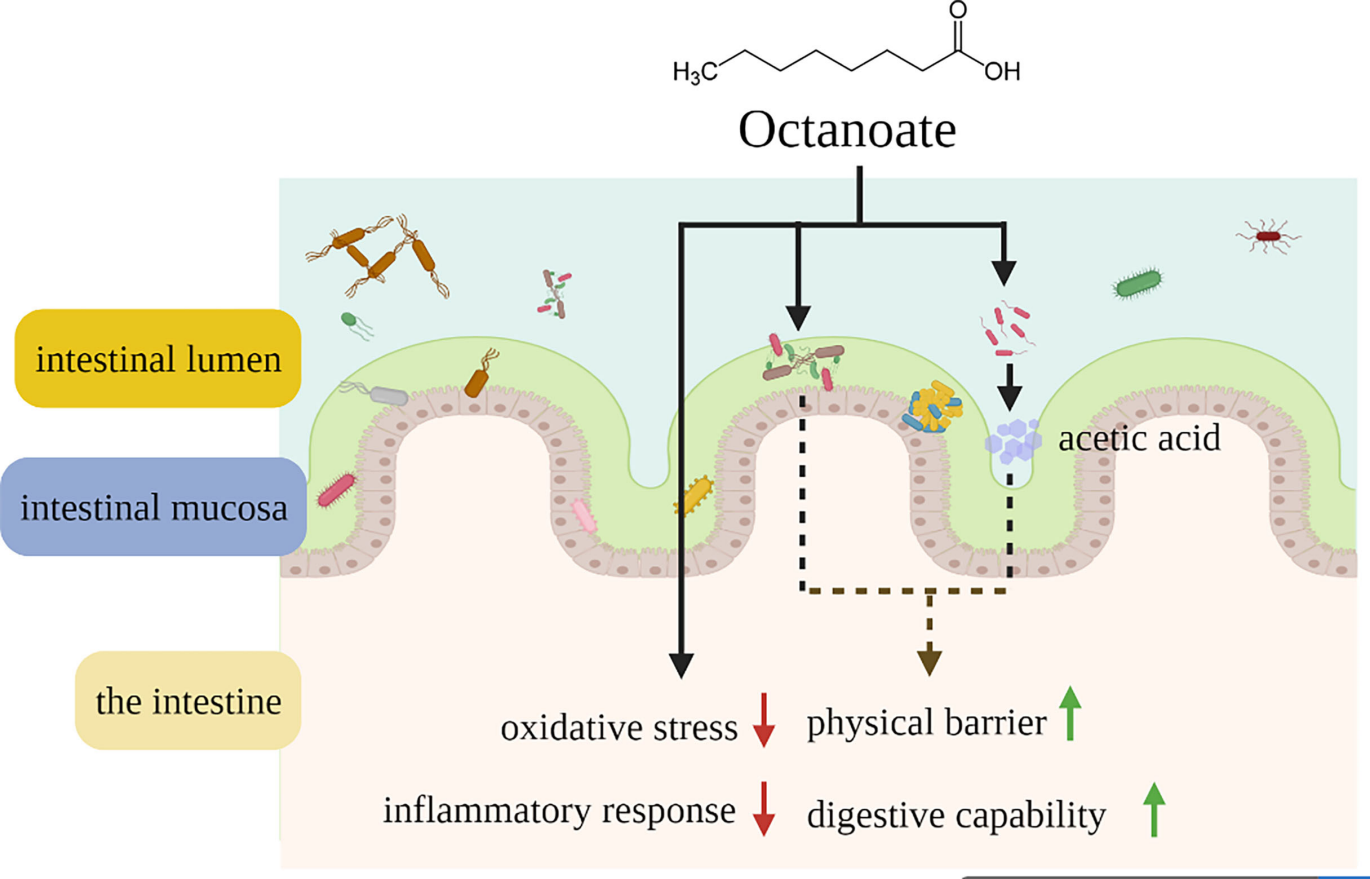
Schematic representation showing that dietary octanoate repaired intestinal physical barrier damage, oxidative stress, inflammatory response and microbial dysbiosis.

In this study, we demonstrated the specific role of octanoate in repairing intestinal damage for the first time. Dietary octanoate might alleviated intestinal oxidative stress, inflammatory response, and microbial dysbiosis to repair intestinal damage caused by dietary SO stress in large yellow croaker at a concentration range of 2.1-6.3 g/kg (**Figure 6**). This study supported the utility of octanoate as an alternative adjuvant therapeutic agented for intestinal diseases and microbial dysbiosis, and these findings might be helpful to improve vertebrate health.

## Data Availability Statement

The datasets presented in this study can be found in online repositories. The names of the repository/repositories and accession number(s) can be found below: https://www.ncbi.nlm.nih.gov/, PRJNA814750.

## Ethics Statement

The animal study was reviewed and approved by Standard Operation Procedures of the Institutional Animal Care and Use Committee of the Ocean University of China.

## Author Contributions

ZZ and QA: Designed the experiments, performed the main experiments, and wrote the manuscript. YT and GL: Conducted other experiments. WF and DX: Analyzed and interpreted the data. KC, SC, BT, and KM: revised the manuscript. QA: Conceptualized, Wrote - reviewed & edited, Supervision. All authors contributed to the final editing and approval of the manuscript.

## Funding

This research is supported by Key Program of National Natural Science Foundation of China [grant number: 31830103], China Agriculture Research System of MOF and MARA(CARS-47), the National Science Fund for Distinguished Young Scholars of China [grant number: 31525024], Scientific and Technological Innovation of Blue Granary [grant number: 2018YFD0900402].

## Conflict of Interest

The authors declare that the research was conducted in the absence of any commercial or financial relationships that could be construed as a potential conflict of interest.

## Publisher’s Note

All claims expressed in this article are solely those of the authors and do not necessarily represent those of their affiliated organizations, or those of the publisher, the editors and the reviewers. Any product that may be evaluated in this article, or claim that may be made by its manufacturer, is not guaranteed or endorsed by the publisher.

## Supplementary Material

Supplementary Table 1 Formulation and chemical proximate analysis of the experimental diets (% dry weight) ^a^ All ingredients were purchased from Great Seven Biotechnology Co., Ltd, China. ^b^ White fish meal (dry mater, %): 74% crude protein and 12.6% crude lipid; Krill meal (dry mater, %): 53.18% crude protein and 13% crude lipid; Bean meal (dry mater, %): 50.08% crude protein and 0.35% crude lipid; High-gluten flour (dry mater, %): 21.2% crude protein and 0.34% crude lipid. ^c^ The mixture of mineral and vitamin, antioxidant, attractant mixture, and mildew preventive according to (19).

Supplementary Table 2 Fatty acid profiles of the experimental diets (% total fatty acids) ^a^ SFAs, saturated fatty acids; MUFAs, monounsaturated fatty acids; PUFAs, polyunsaturated fatty acids; n-3/n-6 PUFA, the ratio of n-3 PUFA to n-6 PUFA. ^b^ Other FAs, some fatty acids of which the contents are minor trace amount or not detected, such as C22: 0, C24: 0, C14: 1n-6, C20: 2n-6, C20:3n-6, were not listed in the table. ^c^ Total FAs, all fatty acids in diets. ^d^ ND, Not found in samples.

Supplementary Table 3 Sequences of the RT-qPCR primers used in this study. ZO-1: Zonula Occludens-1; ZO-2: Zonula Occludens-2; CAT: catalase; Nrf2: nuclear factor erythroid 2-related factor; SOD: superoxide dismutase; IL-1β: interleukin-1β; IL-6: interleukin-6; IL-8: interleukin-8; TNF-α: tumour necrosis factor α; IFN-γ: interferon γ; iNOS: inducible nitric oxide synthase; MHC: major histocompatibility complex; Arg1: arginase 1; IL-10: interleukin-10; CD68: cluster of differentiation 68; CD209: cluster of differentiation 209; MRC1: macrophage mannose receptor 1;TLR: toll-like receptor; MyD88: myeloid differentiation factor 88.

Supplementary Table 4 Fatty acid profiles (% total fatty acids) in intestinal tissue of large yellow croaker (*Larmichthys crocea*) fed the experimental diets (Means ± S.E.M) ^d^. ^a^ SFAs, saturated fatty acids; MUFAs, monounsaturated fatty acids; PUFAs, polyunsaturated fatty acids; n-3/n-6PUFA, the ratio of n-3 PUFAs to n-6 PUFAs. ^b^ Other FAs, some fatty acids of which the contents are minor trace amount or not detected, such as C22: 0, C24: 0, C14: 1n-6, C20: 2n-6, C20:3n-6, were not listed in the table. ^c^ Total FAs, all fatty acids in diets. ^d^ Data were presented as means ± S.E.M. Means in each row sharing the same superscript letter or absence of superscripts were not significantly different determined by Tukey's test (P ≥ 0.05). S.E.M.: standard error of means. ^e^ ND, Not found in samples.

Supplementary Table 5 The list of abbreviations.

Supplementary Figure 1 The protocol of the experiment. Intestinal damage of large yellow croaker was induced by 70 g/kg SO in the diet for 10 weeks feeding experiment. Different concentrations of sodium OCT were intervened, and collected the samples after feeding experiment.

Supplementary Figure 2 The per-experiments of exploring the appropriate concentration of DHA and LA to simulate the situation *in vivo* **(A, B)**. The relative mRNA expression of key antioxidation-related genes (*cat*, *nrf2*, *sod1*, and *sod2*) **(A)** and the fold change of ROS in intestinal cells **(B)**. The evaluation of sequencing efforts’ completeness and depth **(C–F)**. Both the rarefaction curve **(C)** and species accumulation boxplot **(D)** of mucosal microbiota, tended to approach the saturation plateau. The rarefaction curve **(E)** and species accumulation boxplot **(F)** of content flora both tended to approach the saturation plateau (n=4). The profile of content flora (n=4) **(G–O)**. The histogram of relative abundance of phylum **(G)** and genus **(H)** (Only top 10 most abundant (based on relative abundance) bacterial phyla and genera were shown. Other phyla and genera were all assigned as ‘Others’.) were calculated to portray the fundamental structure of content flora. The ratio of Firmicute to Bacteroidetes (n=3) **(I)** was calculated to portray the fundamental structure of gut content flora. Alpha diversity indices included the Simpson index **(J)**, the Shannon index **(K)**, the Chao 1 index **(L)**, and the abundance-based coverage estimator (Ace) index **(M)** were calculated to compare the community diversity and richness. The beta diversity analyses were performed on the weighted Unifrac distance matrix to analyze the extent of similarities in microbial communities, including the principal coordinates analysis (PCoA) **(N)** and UPGMA clustering **(O)**. Data of the relative mRNA of key antioxidation-related genes and the fold change of ROS were presented as means ± S.E.M. Means in each bar sharing the same superscript letter or absence of superscripts were not significantly different determined by Tukey’s test (*P* ≥ 0.05). S.E.M.: standard error of means (n=3).

## References

[1] Ye Z Xu YJ Liu YF. Influences of Dietary Oils and Fats, and the Accompanied Minor Content of Components on the Gut Microbiota and Gut Inflammation: A Review. Trends Food Sci Technol (2021) 113:255–76. doi: 10.1016/j.tifs.2021.05.001

[2] Shores DR Binion DG Freeman BA Baker PR. New Insights Into the Role of Fatty Acids in the Pathogenesis and Resolution of Inflammatory Bowel Disease. Inflammation Bowel Dis (2011) 17(10):2192–204. doi: 10.1002/ibd.21560 PMC4100336 21910181

[3] Investigators IBDiES Tjonneland A Overvad K Bergmann MM Nagel G Linseisen J. Linoleic Acid, a Dietary N-6 Polyunsaturated Fatty Acid, and the Aetiology of Ulcerative Colitis: A Nested Case-Control Study Within a European Prospective Cohort Study. Gut (2009) 58(12):1606–11. doi: 10.1136/gut.2008.169078 19628674

[4] Vancamelbeke M Vermeire S. The Intestinal Barrier: A Fundamental Role in Health and Disease. Expert Rev Gastroenterol Hepatol (2017) 11(9):821–34. doi: 10.1080/17474124.2017.1343143 PMC6104804 28650209

[5] Gulhane M Murray L Lourie R Tong H Sheng YH Wang R. High Fat Diets Induce Colonic Epithelial Cell Stress and Inflammation That Is Reversed by Il-22. Sci Rep (2016) 6:17. doi: 10.1038/srep28990 27350069 PMC4924095

[6] Schonfeld P Wojtczak L. Short- and Medium-Chain Fatty Acids in Energy Metabolism: The Cellular Perspective. J Lipid Res (2016) 57(6):943–54. doi: 10.1194/jlr.R067629 PMC4878196 27080715

[7] Augustin K Khabbush A Williams S Eaton S Orford M Cross JH. Mechanisms of Action for the Medium-Chain Triglyceride Ketogenic Diet in Neurological and Metabolic Disorders. Lancet Neurol (2018) 17(1):84–93. doi: 10.1016/s1474-4422(17)30408-8 29263011

[8] Cansiz D Unal I Ustundag UV Alturfan AA Altinoz MA Elmaci I. Caprylic Acid Ameliorates Rotenone Induced Inflammation and Oxidative Stress in the Gut-Brain Axis in Zebrafish. Mol Biol Rep (2021). doi: 10.1007/s11033-021-06532-5 34228274

[9] Zhang X Xue C Xu Q Zhang Y Li H Li F. Caprylic Acid Suppresses Inflammation *Via* Tlr4/Nf-Kappab Signaling and Improves Atherosclerosis in Apoe-Deficient Mice. Nutr Metab (Lond) (2019) 16:40. doi: 10.1186/s12986-019-0359-2 31182969 PMC6555760

[10] Marounek M Putthana V Benada O Lukesova D. Antimicrobial Activities of Medium-Chain Fatty Acids and Monoacylglycerols on *Cronobacter Sakazakii* Dbm 3157(T) and *Cronobacter Malonaticus* Dbm 3148. Czech J Food Sci (2012) 30(6):573–80. doi: 10.17221/433/2011-cjfs

[11] Wang JY Ma MM Yang J Chen L Yu P Wang J. *In Vitro* Antibacterial Activity and Mechanism of Monocaprylin Against *Escherichia Coli* and *Staphylococcus Aureus* . J Food Prot (2018) 81(12):1988–96. doi: 10.4315/0362-028x.jfp-18-248 30461297

[12] Zhang JH Feng FQ Zhao MJ. Glycerol Monocaprylate Modulates Gut Microbiota and Increases Short-Chain Fatty Acids Production Without Adverse Effects on Metabolism and Inflammation. Nutrients (2021) 13(5):18. doi: 10.3390/nu13051427 PMC8147114 33922631

[13] Liu W Luo X Tang J Mo Q Zhong H Zhang H. A Bridge for Short-Chain Fatty Acids to Affect Inflammatory Bowel Disease, Type 1 Diabetes, and Non-Alcoholic Fatty Liver Disease Positively: By Changing Gut Barrier. Eur J Nutr (2020). doi: 10.1007/s00394-020-02431-w 33180143

[14] Fan Y Pedersen O. Gut Microbiota in Human Metabolic Health and Disease. Nat Rev Microbiol (2021) 19(1):55–71. doi: 10.1038/s41579-020-0433-9 32887946

[15] Jia M Zhang Y Gao Y Ma X. Effects of Medium Chain Fatty Acids on Intestinal Health of Monogastric Animals. Curr Protein Pept Sci (2020) 21(8):777–84. doi: 10.2174/1389203721666191231145901 31889482

[16] Chen Q Du J Cui K Fang W Zhao Z Chen Q. Acetyl-Coa Derived From Hepatic Mitochondrial Fatty Acid β-Oxidation Aggravates Inflammation by Enhancing P65 Acetylation. iScience (2021) 24(11). doi: 10.1016/j.isci.2021.103244 PMC8551082 34746707

[17] Du J Xiang X Li Y Ji R Xu H Mai K. Molecular Cloning and Characterization of Farnesoid X Receptor From Large Yellow Croaker (*Larimichthys Crocea*) and the Effect of Dietary Cdca on the Expression of Inflammatory Genes in Intestine and Spleen. Comp Biochem Physiol B Biochem Mol Biol (2018) 216:10–7. doi: 10.1016/j.cbpb.2017.09.007 28982586

[18] Ai Q Mai K Tan B Xu W Zhang W Ma H. Effects of Dietary Vitamin C on Survival, Growth, and Immunity of Large Yellow Croaker. Pseudosciaena Crocea. Aquacult (2006) 261(1):327–36. doi: 10.1016/j.aquaculture.2006.07.027

[19] Li X Ji R Cui K Chen Q Chen Q Fang W. High Percentage of Dietary Palm Oil Suppressed Growth and Antioxidant Capacity and Induced the Inflammation by Activation of Tlr-Nf-Kappab Signaling Pathway in Large Yellow Croaker (*Larimichthys Crocea*). Fish Shellfish Immunol (2019) 87:600–8. doi: 10.1016/j.fsi.2019.01.055 30738147

[20] Yin Z Liu Q Liu Y Gao S He Y Yao C. Early Life Intervention Using Probiotic Clostridium Butyricum Improves Intestinal Development, Immune Response, and Gut Microbiota in Large Yellow Croaker (*Larimichthys Crocea*) Larvae. Front Immunol (2021) 12:640767. doi: 10.3389/fimmu.2021.640767 33763082 PMC7982665

[21] Cui K Li X Chen Q Li Q Gao S Tan P. Effect of Replacement of Dietary Fish Oil With Four Vegetable Oils on Prostaglandin E2 Synthetic Pathway and Expression of Inflammatory Genes in Marine Fish *Larimichthys Crocea* . Fish Shellfish Immunol (2020) 107(Pt B):529–36. doi: 10.1016/j.fsi.2020.09.038 33152403

[22] Liu Y Miao Y Xu N Ding T Cui K Chen Q. Effects of Dietary Astragalus Polysaccharides (Aps) on Survival, Growth Performance, Activities of Digestive Enzyme, Antioxidant Responses and Intestinal Development of Large Yellow Croaker (*Larimichthys Crocea*) Larvae. Aquaculture (2020) 517. doi: 10.1016/j.aquaculture.2019.734752

[23] Chen Q Fang W Cui K Chen Q Xiang X Zhang J. Endoplasmic Reticulum Stress Induces Hepatic Steatosis by Transcriptional Upregulating Lipid Droplet Protein Perilipin2. FASEB J (2021) 35(10):e21900. doi: 10.1096/fj.202100739RR 34547130

[24] Fang W Chen Q Li J Liu Y Zhao Z Shen Y. Endoplasmic Reticulum Stress Disturbs Lipid Homeostasis and Augments Inflammation in the Intestine and Isolated Intestinal Cells of Large Yellow Croaker (*Larimichthys Crocea*). Front Immunol (2021) 12:738143. doi: 10.3389/fimmu.2021.738143 34489982 PMC8417523

[25] Fang W Chen Q Cui K Chen Q Li X Xu N. Lipid Overload Impairs Hepatic Vldl Secretion Via Oxidative Stress-Mediated Pkcdelta-Hnf4alpha-Mtp Pathway in Large Yellow Croaker (*Larimichthys Crocea*). Free Radic Biol Med (2021). doi: 10.1016/j.freeradbiomed.2021.06.001 34116177

[26] Magoc T Salzberg SL. Flash: Fast Length Adjustment of Short Reads to Improve Genome Assemblies. Bioinformatics (2011) 27(21):2957–63. doi: 10.1093/bioinformatics/btr507 PMC3198573 21903629

[27] Caporaso JG Kuczynski J Stombaugh J Bittinger K Bushman FD Costello EK. Qiime Allows Analysis of High-Throughput Community Sequencing Data. Nat Methods (2010) 7(5):335–6. doi: 10.1038/nmeth.f.303 PMC3156573 20383131

[28] Edgar RC. Uparse: Highly Accurate Otu Sequences From Microbial Amplicon Reads. Nat Methods (2013) 10(10):996. doi: 10.1038/nmeth.2604 23955772

[29] DeSantis TZ Hugenholtz P Larsen N Rojas M Brodie EL Keller K. Greengenes, a Chimera-Checked 16s Rrna Gene Database and Workbench Compatible With Arb. Appl Environ Microbiol (2006) 72(7):5069–72. doi: 10.1128/aem.03006-05 PMC1489311 16820507

[30] Segata N Izard J Waldron L Gevers D Miropolsky L Garrett WS. Metagenomic Biomarker Discovery and Explanation. Genome Biol (2011) 12(6). doi: 10.1186/gb-2011-12-6-r60 PMC3218848 21702898

[31] Tan P Dong X Mai K Xu W Ai Q. Vegetable Oil Induced Inflammatory Response by Altering Tlr-Nf-κb Signalling, Macrophages Infiltration and Polarization in Adipose Tissue of Large Yellow Croaker (*Larimichthys Crocea*). Fish Shellfish Immunol (2016) 59:398–405. doi: 10.1016/j.fsi.2016.11.009 27818336

[32] Rios-Covian D Ruas-Madiedo P Margolles A Gueimonde M de los Reyes-Gavilan CG Salazar N. Intestinal Short Chain Fatty Acids and Their Link With Diet and Human Health. Front Microbiol (2016) 7:185. doi: 10.3389/fmicb.2016.00185 26925050 PMC4756104

[33] Konig J Wells J Cani PD Garcia-Rodenas CL MacDonald T Mercenier A. Human Intestinal Barrier Function in Health and Disease. Clin Transl Gastroenterol (2016) 7:13. doi: 10.1038/ctg.2016.54 PMC5288588 27763627

[34] Tian T Wang Z Zhang J. Pathomechanisms of Oxidative Stress in Inflammatory Bowel Disease and Potential Antioxidant Therapies. Oxid Med Cell Longev (2017) 2017:4535194. doi: 10.1155/2017/4535194 28744337 PMC5506473

[35] Ungaro R Mehandru S Allen PB Peyrin-Biroulet L Colombel J-F. Ulcerative Colitis. Lancet (2017) 389(10080):1756–70. doi: 10.1016/s0140-6736(16)32126-2 PMC6487890 27914657

[36] Torres J Mehandru S Colombel J-F Peyrin-Biroulet L. Crohn's Disease. Lancet (2017) 389(10080):1741–55. doi: 10.1016/s0140-6736(16)31711-1 27914655

[37] Chwen LT Foo HL Nguyen Tien T Choe DW. Growth Performance, Plasma Fatty Acids, Villous Height and Crypt Depth of Preweaning Piglets Fed With Medium Chain Triacylglycerol. Asian-Australas J Anim Sci (2013) 26(5):700–4. doi: 10.5713/ajas.2012.12561 PMC4093331 25049841

[38] Zhao J Hu J Ma X. Sodium Caprylate Improves Intestinal Mucosal Barrier Function and Antioxidant Capacity by Altering Gut Microbial Metabolism. Food Funct (2021) 12(20):9750–62. doi: 10.1039/d1fo01975a 34664601

[39] Zhong W Li Q Xie G Sun X Tan X Sun X. Dietary Fat Sources Differentially Modulate Intestinal Barrier and Hepatic Inflammation in Alcohol-Induced Liver Injury in Rats. Am J Physiol Gastrointest Liver Physiol (2013) 305(12):G919–32. doi: 10.1152/ajpgi.00226.2013 PMC3882440 24113767

[40] Yue CH Li M Li J Han X Zhu HW Yu GP. Medium-, Long- and Medium-Chain-Type Structured Lipids Ameliorate High-Fat Diet-Induced Atherosclerosis by Regulating Inflammation, Adipogenesis, and Gut Microbiota in Apoe^(^-^/-)^Mice. Food Funct (2020) 11(6):5142–55. doi: 10.1039/d0fo01006e 32432606

[41] Yu S Go GW Kim W. Medium Chain Triglyceride (Mct) Oil Affects the Immunophenotype *Via* Reprogramming of Mitochondrial Respiration in Murine Macrophages. Foods (2019) 8(11). doi: 10.3390/foods8110553 PMC6915711 31694322

[42] Roda G Chien Ng S Kotze PG Argollo M Panaccione R Spinelli A. Crohn's Disease. Nat Rev Dis Primers (2020) 6(1):22. doi: 10.1038/s41572-020-0156-2 32242028

[43] Morgan XC Tickle TL Sokol H Gevers D Devaney KL Ward DV. Dysfunction of the Intestinal Microbiome in Inflammatory Bowel Disease and Treatment. Genome Biol (2012) 13(9). doi: 10.1186/gb-2012-13-9-r79 PMC3506950 23013615

[44] Coenye T Spilker T Reik R Vandamme P Lipuma JJ. Use of Pcr Analyses to Define the Distribution of *Ralstonia* Species Recovered From Patients With Cystic Fibrosis. J Clin Microbiol (2005) 43(7):3463–6. doi: 10.1128/JCM.43.7.3463-3466.2005 PMC1169115 16000479

[45] Wang Y Hosomi K Shimoyama A Yoshii K Yamaura H Nagatake T. Adjuvant Activity of Synthetic Lipid a of , a Gut-Associated Lymphoid Tissue-Resident Commensal Bacterium, to Augment Antigen-Specific Igg and Th17 Responses in Systemic Vaccine. Vaccines (Basel) (2020) 8(3). doi: 10.3390/vaccines8030395 PMC7565795 32698404

[46] Leite FL Abrahante JE Vasquez E Vannucci F Gebhart CJ Winkelman N. A Cell Proliferation and Inflammatory Signature Is Induced by *Lawsonia* Intracellularis Infection in Swine. Mbio (2019) 10(1). doi: 10.1128/mBio.01605-18 PMC6355989 30696739

[47] Xu Y Zhu Y Li X Sun B. Dynamic Balancing of Intestinal Short-Chain Fatty Acids: The Crucial Role of Bacterial Metabolism. Trends Food Sci Technol (2020) 100:118–30. doi: 10.1016/j.tifs.2020.02.026

[48] Takeuchi T Miyauchi E Kanaya T Kato T Nakanishi Y Watanabe T. Acetate Differentially Regulates Iga Reactivity to Commensal Bacteria. Nature (2021). doi: 10.1038/s41586-021-03727-5 34262176

[49] Li M Hu FC Qiao F Du ZY Zhang ML. Sodium Acetate Alleviated High-Carbohydrate Induced Intestinal Inflammation by Suppressing Mapk and Nf-Kappa B Signaling Pathways in Nile Tilapia (*Oreochromis Niloticus*). Fish Shellfish Immunol (2020) 98:758–65. doi: 10.1016/j.fsi.2019.11.024 31730927

