# Supplementary figures and images for: Octanoate Alleviates Dietary Soybean Oil-Induced Intestinal Physical Barrier Damage, Oxidative Stress, Inflammatory Response and Microbial Dysbiosis in Large Yellow Croaker (Larimichthys Crocea)
Source: Front Immunol. 2022 Jun 29;13:892901. doi: 10.3389/fimmu.2022.892901 (PMC9277137; doi:10.3389/fimmu.2022.892901)

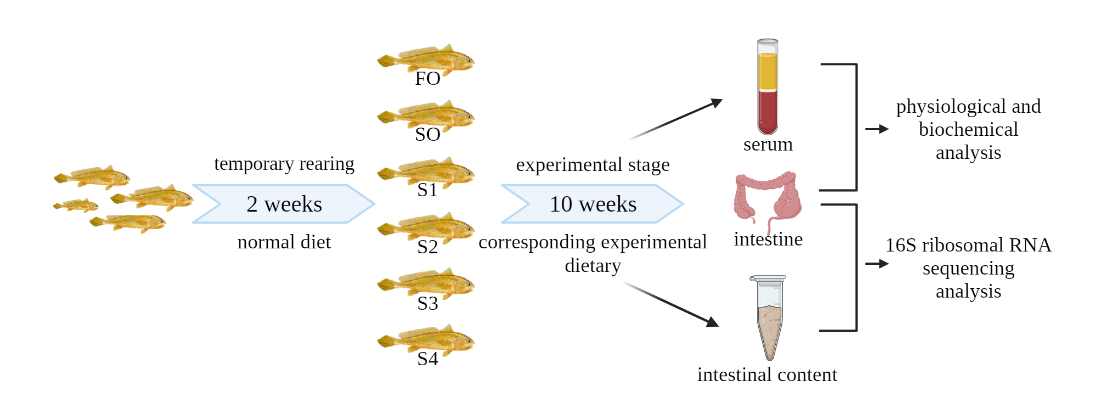

Supplement: Supplementary Figure 1 — The protocol of the experiment. Intestinal damage of large yellow croaker was induced by 70 g/kg SO in the diet for 10 weeks feeding experiment. Different concentrations of sodium OCT were intervened, and collected the samples after feeding experiment. [file Image_1.tif]

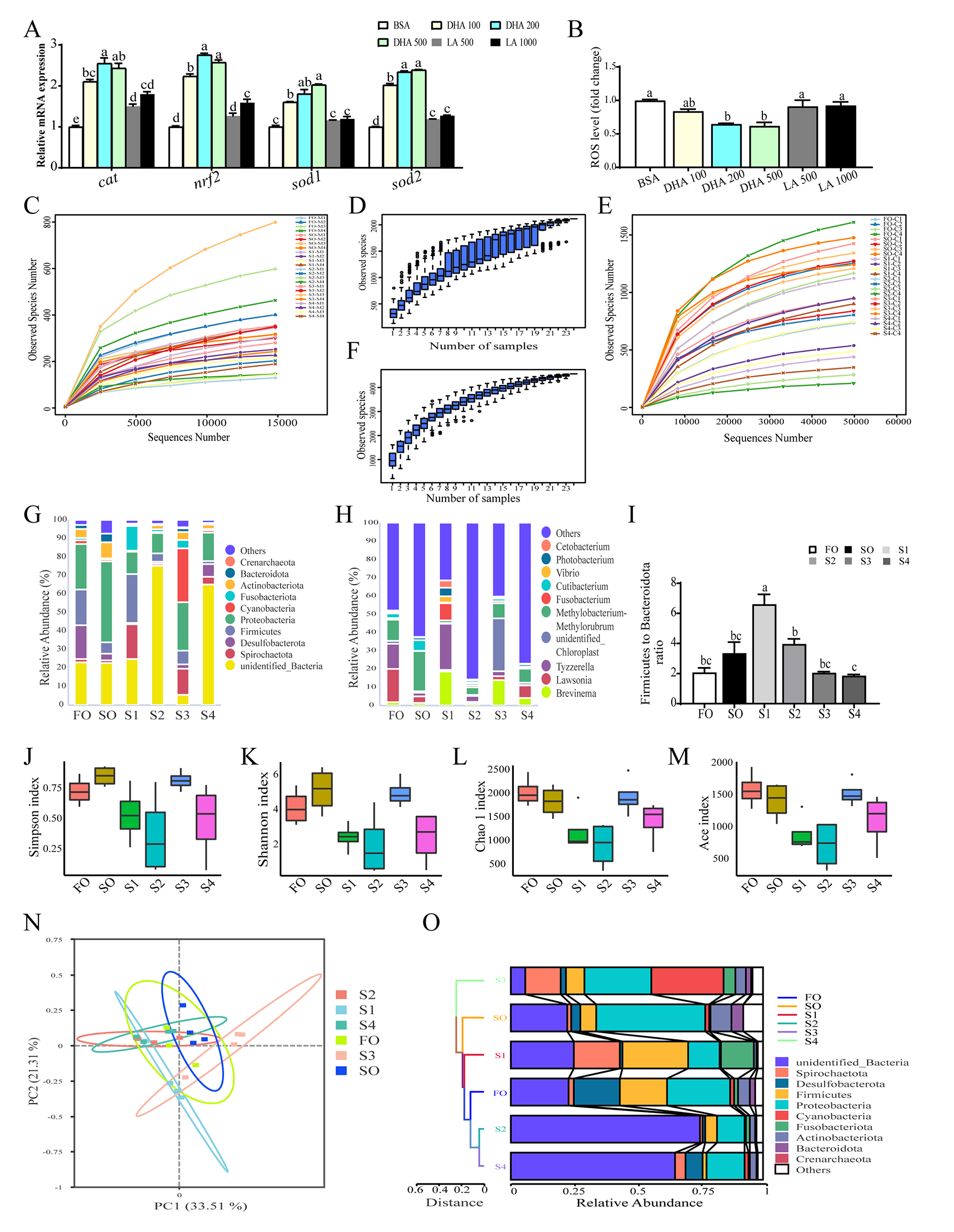

Supplement: Supplementary Figure 2 — The per-experiments of exploring the appropriate concentration of DHA and LA to simulate the situation in vivo (A, B). The relative mRNA expression of key antioxidation-related genes (cat, nrf2, sod1, and sod2) (A) and the fold change of ROS in intestinal cells (B). The evaluation of sequencing efforts’ completeness and depth (C–F). Both the rarefaction curve (C) and species accumulation boxplot (D) of mucosal microbiota, tended to approach the saturation plateau. The rarefaction curve (E) and species accumulation boxplot (F) of content flora both tended to approach the saturation plateau (n=4). The profile of content flora (n=4) (G–O). The histogram of relative abundance of phylum (G) and genus (H) (Only top 10 most abundant (based on relative abundance) bacterial phyla and genera were shown. Other phyla and genera were all assigned as ‘Others’.) were calculated to portray the fundamental structure of content flora. The ratio of Firmicute to Bacteroidetes (n=3) (I) was calculated to portray the fundamental structure of gut content flora. Alpha diversity indices included the Simpson index (J), the Shannon index (K), the Chao 1 index (L), and the abundance-based coverage estimator (Ace) index (M) were calculated to compare the community diversity and richness. The beta diversity analyses were performed on the weighted Unifrac distance matrix to analyze the extent of similarities in microbial communities, including the principal coordinates analysis (PCoA) (N) and UPGMA clustering (O). Data of the relative mRNA of key antioxidation-related genes and the fold change of ROS were presented as means ± S.E.M. Means in each bar sharing the same superscript letter or absence of superscripts were not significantly different determined by Tukey’s test (P ≥ 0.05). S.E.M.: standard error of means (n=3). [file Image_2.tif]
